# Surgery

**Published:** 1861-05

**Authors:** S. W. Gross

**Affiliations:** Lecturer on Surgical Anatomy and Operative Surgery, Philadelphia


### Bi-Monthly Abstracts;

OR, BI-MONTHLY NOVELTIES IN MEDICAL, PATHOLOGICAL, SURGICAL, OBSTETRICAL, PHYSIOLOGICAL, AND CHEMICAL SCIENCE.

### SURGERY.

BY S. W. GROSS, M.D.,

LECTURER ON SURGICAL ANATOMY AND OPERATIVE SURGERY, PHILADELPHIA.

### I.—*Contributions to the Subject of Compound Fracture.* By Mr. Thomas Bryant, F.R.C.S. (Medical Times and Gazette, Feb. 23, 1861.)

In this paper, read before the Royal Medical and Chirurgical Society of London, Mr. Bryant gives an analysis of 302 cases of compound fracture of the extremities, taken from the records of Guy's Hospital within the last twenty years. Excluding the examples of fracture of the smaller bones of the hands and feet, we find that the whole number includes 17, or 5·6 per cent., of the thigh; 193, or 63·9 per cent., of the leg; 35, or 11·5 per cent., of the arm; and 57, or 18·8 per cent., of the forearm. Taking up the classes of cases in the order above given, we find:—

1. That eleven of the seventeen cases of compound fracture of the thigh proved fatal, or 64·7 per cent. Nine of these examples underwent primary amputation, of which but three recovered. One underwent secondary amputation, death being the result. Seven were treated on conservative principles; four died, and three recovered. Ten were, therefore, subjected to amputation, and seven died; seven were left to the efforts of nature, and four of these died. This result shows the poor success obtained by conservative treatment, and is borne out by the experience of most military surgeons. Baudens succeeded in saving but two out of twenty-five cases, and these had useless and deformed limbs; and the author would, therefore, leave to nature's efforts at repair those cases only in which the subjects are young, the fractures uncomplicated, the bone not shattered, nor the soft parts extensively injured. Amputation should be at once performed in old or unhealthy patients, when the bone was comminuted and the soft parts materially involved; but in young and healthy subjects a favorable termination might be obtained by immediately removing the fragments, and maintaining absolute rest.

2. The 193 cases of compound fracture of the leg include fractures of the tibia, of the tibia and fibula combined, these being the most numerous, and of the fibula alone, but this injury was comparatively rare. Of the whole number seventy-four died, or 38·3 per cent. Conservative treatment was employed in 129, thirty-five proving fatal; thirty-two underwent primary amputation of the leg, nineteen of which died; eleven underwent primary amputation of the thigh, six of which died; fifteen underwent secondary amputation of the leg, ten proving fatal; and six underwent secondary amputation of the thigh, four of which died. Thus we find that 27·13 per cent. of those dying were treated by conservative measures, and 60·9 per cent. of those treated by amputation. Mr. Bryant believes that the injury should be very extensive to warrant amputation, and that apparently hopeless cases often turn out well, when attempts are made to save the limb. By care and close attention, support and nourishment, giving free exit to pus, removing loose pieces of bone as early as possible, and the use of proper splints to secure perfect rest, successful results may be looked for.

3. Of the thirty-five cases of compound fracture of the arm, four proved fatal. Fourteen were treated on conservative principles, and all recovered; four were subjected to primary amputation at the shoulder-joint, and two died from internal complications; thirteen were treated by primary amputation, two of which died; four were treated by secondary amputation, and recovered.

4. Twenty-seven of the fifty-seven cases of compound fracture of the bones of the forearm were treated on conservative measures, and all recovered; twenty-two were treated by primary amputation of the forearm, of which two died; five were treated by secondary amputation, four of the forearm, one died; one of the arm, which proved fatal.

The causes of death in the ninety-six fatal examples are given in a table, from which it appears that pyæmia was twice as fatal in the cases subjected to amputation as in those treated on ordinary surgical conservative principles; and that exhaustion or gangrene were the more common causes of death. Delirium tremens and tetanus were the most prolific sources of a fatal result in the cases left to nature's efforts for repair.

II.—*Statistics of Compound Fracture at St. George's Hospital.* By MR. G. F. COOPER. (Medical Times and Gazette, Feb. 16, 1861.)

During a period of seven years, 124 cases of compound fracture have been admitted into St. George's Hospital, of which thirty-five died, or 28·2 per cent. The thigh was the seat of the accident in sixteen instances, of which eight died; the leg in eighty-two, of which twenty-three died; the arm in eleven, of which three died; the forearm in sixteen, of which one died. Primary amputation was resorted to in twenty-three cases, nine being fatal. Of these, seven were of the thigh, of which four died; eight of the leg, of which five died; four of the arm, and four of the forearm, of which all recovered. Secondary amputation was had recourse to in five instances, four being of the leg, with one death, and one of the forearm, which recovered.

In regarding the age of the patients we find a gradual increase of mortality with advance of life. Thus in infancy there were two cases, and both recovered; in childhood there were seventeen, of which two died; in adults there were seventy-two, of which nineteen died; from fifty years of age upwards there were thirty-three, of which fourteen died. These results show that age is a very important point to be considered in the treatment, and the influence it bears on the mortality of compound fractures.

In twenty-four of the whole number of cases the compound fracture communicated with a joint. Seven involved the knee, of which three recovered, the average time in the hospital being eighty days. In all but one primary amputation was performed. Of the ankle-joint there were nine cases, of which six recovered, the average time in the hospital being ninety-one days. Primary amputation was resorted to in one of the cases that recovered, and in two of those that died. There were eight cases of the elbow-joint, five of which recovered, the average time in the hospital being eighty-nine days. In one case of recovery, and in one of death, primary amputation was had recourse to.

Of the thirty-five fatal cases, the causes of death were principally other severe injuries, pyæmia, and exhaustion.

III.—*Clinical Experience in the Treatment of Stone, during the Year* 1860. By Dr. Civiale. (Lancet, March 2, 1861.)

During the past year fifty-four patients affected with urinary calculus have been under the charge of Dr. Civiale, thirty-six occurring in his private practice, and eighteen in the Necker Hospital. Of the *private patients* twenty-six had stone for the first time, and in ten others fresh operations were required, on account of the stone having reappeared after previous operations. Twenty-six of these patients were subjected to lithotrity, and twenty-four were cured; in two the operation had to be relinquished from aggravation of the morbid condition of the bladder. One patient has since died, and the other still lives with the calculus remaining. In ten cases, the stone had acquired considerable volume and the urinary organs were in a more or less morbid condition. In two of these lithotrity and lithotomy being equally contra-indicated, death ensued from the progress of the malady. Four were lithotomized on account of the large size of the calculus, of which one rapidly recovered; in one the cure was incomplete, and two died. Two are still under treatment: one will be operated on by lithotrity and one by lithotomy; and in two the treatment has been postponed.

*The hospital patients* numbered eighteen, three being women, and all adults. Of the males four were not in a condition for crushing; two were cut, one being cured, but the other has a fistule; the third died of renal disease, refusing to submit to the knife, and the fourth is under treatment. One patient was also the subject of a strangulated hernia, requiring immediate operation, from which he died. The remaining ten were submitted to lithotrity and freed from the calculus. Eight of those recovered perfectly, but in two the cure was not complete, owing to organic lesions of the bladder. Of the females one was in so bad a condition that an operation was not performed; one was cured by division of the urethra and extraction of the stone; and the third had an extraordinary phosphatic calculus, formed upon a mass of teeth, of small bones, and of hair, arising from a cyst which had opened into the bladder. All these substances were gotten rid of by lithotrity.

M. Civiale terminates his communication with the following resumé:—

Of the fifty-four calculous patients, thirty-seven have been treated by lithotrity. In two cases the treatment was relinquished; one died; another retains his stone; two of them have not obtained a complete cure, because the stone has not formed the sole malady, but they are greatly relieved. The rest are cured.

Seven were submitted to lithotomy which saved four of them; but in two of these the cure is incomplete.

Ten have not been submitted to any operation; three have died from the progress of the malady, and one after the operation for hernia; one continues to live with his stone. Three are under treatment, and will be submitted, one to lithotrity, and two to lithotomy. In two cases the operations are postponed.

These facts prove anew the danger of long retaining the stone, and the utility of lithotrity when we apply it at an early period of the complaint.

IV.—*Case of Severe Injury of the Head, with Loss of Brain.* By W. H. MATCHETT, M.D. (Cleveland Medical Gazette, February, 1861.)

Dr. Matchett was called on the 9th of January, 1860, to see a man whom he found sitting by the stove, his face and clothing covered with blood, and frequently vomiting blood. He was not sensible of any particular injury, merely complaining of some pain in his shoulder and arm, on examining which, an axillary dislocation of the humerus was discovered. The head of the bone readily slipped into place by simply carrying the arm out from the body, and on inspecting the head and face more closely, Dr. Matchett "found the upper lip cut entirely through, from one angle of the mouth to the other: a small opening under the left eye, that was bleeding profusely, also a small opening in the centre of the frontal bone—a cut about one and a half inches long, extending from the outer extremity of the right superciliary ridge upward and outward into the temporal fossa. Through this opening, at every effort to vomit or to clear the nostrils, small fragments of brain would escape. I collected these until I had, as near as I could guess, about one-half ounce, and how much more had escaped before I saw him, I cannot say."

"Upon further examination I found the skull fractured from about the middle third of the left superciliary ridge, upward and to the centre of the frontal bone; thence toward the right to the temporal fossa; thence toward the outer extremity of the right superciliary ridge—the latter entirely broken down; and this circumscribed or detached piece of the frontal broken up into four smaller fragments. The nasal bones were driven in from their attachment; the malars broken and movable; the superior maxilla driven downward, and six of the front teeth of the same broken off even with the gums. Four front teeth of the lower jaw were pressed backward, splitting the alveolar process."

The patient was questioned as to how he received the injury, but contended that he was not hurt, his nose merely being stopped up from a severe cold. It was, however, ascertained that he left his house on the same day to fell a tree, and on visiting the spot, it was discovered that he had been injured by a large, knotted limb. By this was found a pool of blood, from which the patient could be tracked to the house, a distance of two hundred yards, having climbed three fences and opened and shut a gate on the route. The cut on the lip was brought together by stitches, and cold compresses were applied to the face and head. He sat in a chair nearly all night, as the recumbent posture caused nausea, and he vomited a quantity of blood. On the second day a cathartic was administered, and a consultation being held, it was decided very naturally that the patient could not live, and no attempt was made to elevate the depressed portions of the frontal bone, or to remove any of the fragments. On the twelfth day, the lip had united, and in attempting to remove the roots of the upper incisors, which caused severe pain, Dr. Matchett found the bones of the face movable, and had, therefore, to desist. The condition of the patient gradually improved, and he had no symptoms of compression. Every effort at blowing the nose would force air into the cellular tissue of the forehead, and for three weeks blood and mucus escaped from the nostrils.

On the first of March, "the nasal, malar, and superior maxillary bones, and the restored teeth of the lower jaw, are all firmly united to their attachments; but the front part of the superior maxilla is too long, allowing the front teeth or roots of the upper and lower teeth, to come together before the molars do, thus preventing the patient from masticating solid food. I found the fragments of the frontal bone united to one another and to the surroundings, but a little depressed. The patient is rational, and remembers shooting a pigeon on the day he was hurt, and also recollects going to the ash tree; but does not remember cutting it down, or making his way to the house, or anything connected with the accident."

The patient continued to do well, and was seen the following September at work on his farm. At the time of writing the report of the case, the author had not heard of any derangement of mind from the loss of brain.

V.—*Constant Success of Forcible Dilatation of the Sphincter, in the Treatment of Fissure of the Anus.* By M. Robert. (Glasgow Medical Journal, January, 1861.)

In cases of fissure of the anus, an affection usually attended with excruciating pain, and indefinitely protracted by spasmodic contraction of the sphincter muscle, M. Robert, contrary to the practice of most surgeons, rejects entirely the operation of division of the muscle, on the ground that it may be followed by serious consequences, as phlebitis, erysipelas, and even death. He considers local applications of slight efficacy, although, in recent cases, he has found the *onguent de la mère*—a combination of lead and basilicon plasters—very beneficial. Instead of these he resorts to forced dilatation, a method introduced into practice by the late Professor Récamier, and in a large number of cases has never failed in effecting a cure, nor has he ever witnessed untoward consequences follow the operation. The method consists in the successive introduction of both forefingers into the anus, which is then forcibly distended in several directions. This procedure elongates and overcomes the contraction of the muscular ring, and the fissure is probably torn and transformed into a simple wound. It is attended with considerable pain, and it, therefore, becomes necessary to place the patient under the influence of chloroform.

VI.—*Popliteal Aneurism Cured by Digital Compression* By George C. Blackman, M.D. (Cincinnati Lancet and Observer, March, 1861.)

A laborer, twenty-seven years of age, applied to Professor Blackman on account of a large popliteal aneurism, of thirteen months' standing, and it was determined to treat the case by the combined methods of compression, manipulation, and flexion. On the 7th of June, 1860, some of the fibrin in the sac was broken up and dislodged, and the limb was enveloped in a roller from the toes to groin, compresses being placed over the tumor and along the course of the femoral artery. The leg was then strongly flexed upon the thigh, and secured in that position. After a week's trial the roller was abandoned and Petit's tourniquet substituted, and at the expiration of another week this was changed for Skey's instrument. Compression was continued for seven days longer, when the patient left for home, the tumor having diminished about one-third in size, but the pulsation being quite distinct. On the first of July, two students applied digital compression at the groin, and in *three hours* pulsation had entirely ceased. Eight months subsequently the man was seen, when he declared that the limb had perfectly recovered its functions. The pulsation had never returned, but the contracted and indurated tumor could still be felt.

VII.—*True Scirrhus of the Eyeball, occurring as a Primary Affection, successfully extirpated; with Special Observations, particularly in Reference to the Operation.* By RICHARD G. H. BUTCHER, ESQ., M.R.I.A. (Dublin Quarterly Journal of Medical Science, February, 1861.)

Many surgeons and pathologists of extended experience are disposed to doubt, and even deny, the existence of scirrhus of the eye, from the fact of never having witnessed an instance of it in this organ; encephaloid and melanosis, but especially the former, being sufficiently common. The case of Mr. Butcher is a well-marked example of this exceedingly rare affection, as is attested by the age of the patient, the peculiar pain, the density and hardness of the tumor, as well as its physical and microscopical appearances.

A woman, sixty-nine years of age, was admitted into Mercer's Hospital, on the 1st of November, 1859, with a tumor, of a circular shape, and about the size of a two-shilling piece, projecting between the lids of the left eye. Its surface was mammillated, and made up of a dense fleshy-colored structure, which, to the touch, was as hard as cartilage, and pressure did not give rise to pain or hemorrhage. A thin watery secretion flowed abundantly from the eye on each side of the growth, and it was the seat of lancinating pain. She was also the subject of severe hemicrania, which was aggravated during the night. Her previous history was that, two years before her admission, she was awakened by a racking pain in the eye, which remained unabated for days, and gradually the sight became dim. For about three months she could discern objects, but the vision was finally lost. Two months later "a red, fleshy pimple" appeared on the cornea, which slowly increased to the size above mentioned.

Mr. Butcher found that the tumor sprung from the cornea and its conjunctival membrane, without implicating the surrounding parts. On the eleventh of November the eye was extirpated, and the case treated in the usual manner. On the fifth of the following month, when she left the hospital, her condition had greatly changed; her appetite was restored, her nights were undisturbed, and she rapidly gained flesh and strength. A careful microscopic investigation of the growth proved it to be of a true scirrhous nature. All the structures, and the whole thickness of the cornea, were matrixed in the abnormal growth. The globe of the eye posterior to its encroachment was not altered in volume; the optic nerve was healthy, as were the sclerotic, and other coats of the organ; the posterior chamber was not encroached upon; the lens lay in its natural situation, while the iris was resting against, but not adherent to it; the anterior chamber was obliterated, in consequence of the cornea being forced backward by the tumor. Every adherent or approximating tissue, as the areolar and muscular, and the lachrymal gland, were subjected to the most searching examination, but were all exempt from contamination.

In commenting upon this remarkable case, the author suggests the following points relative to the operation: 1. On all accounts, it is better to place the patient recumbent, and leaned a little toward the affected side, so that chloroform may be administered to the best advantage, and the safety of the patient; and that the blood may readily escape, and not interfere with the after proceedings of the operation. 2. That the lachrymal gland should be taken away in all instances, when the operation is for cancer, or when an artificial eye is not applicable, so that no undue secretions may be produced, streaming forever over the cheek, the delicate lachrymal puncta being probably obstructed, spoiled in their functions, by the resulting inflammation set up by the injured parts; this objection to its being left being altogether apart from its liability to secondary infection. 3. It is of great importance to the sufferer that no disfiguring traces should remain, by the lids being apart, as so frequently occurs, with a rather prominent red fleshy protrusion from within; or if not projecting, at least appearing sufficiently repulsive within the space.

When an artificial eye cannot be applied, it is necessary to protect the delicate and sensitive parts within from extraneous matters and from sharp winds, and also to secure a placid expression of countenance, free from any trace of deformity. Mr. Butcher suggests for adoption the method of managing the lids pursued in the foregoing case, namely, after separating them by section at the outer canthus, and after the several steps of the operation to the removal of the organ, not bringing the divided surfaces in contact by adhesive strips or stitches, but permitting the upper lid to slightly overlap the lower, by a little more than the depth of its tarsal cartilage; if, as the wound heals, the drooping should threaten to be too great, then the lid may be slightly elevated, which can be readily effected by gently drawing it outward, and maintaining it at the required tension, either by strap or stitch. Sometimes the lid, if redundant, will be inclined to fall inward; this, too, may be prevented by the insertion of a stitch judiciously placed outside, so as to make a little tense the lid, and, at the same time, give it the least possible tendency to eversion of its tarsal edge: when the wound heals, this will not be found too great.

VIII.—*On the Reduction of Luxations of the Head of the Humerus simply by Manipulation.* By HENRY H. SMITH, M.D., Professor of Surgery in the University of Pennsylvania. (Medical and Surgical Reporter, vol. v., No. 19, 1861.)

The method of reducing dislocations of the head of the humerus by elevation and rotation is not a new suggestion, but to Professor Smith is undoubtedly due the credit of having amended and methodized this plan of treatment, and as such to render it worthy the attention of the profession. It does not require the use of an anæsthetic, provided the movements are made gently and slowly, so as not to induce fear or muscular resistance, and may be practiced when the subject is sitting up, in which event the scapula requires to be steadied.

The manipulation for an axillary luxation is as follows: Elevate the humerus as much as possible, or, at least, to a right angle with the body, and flex the forearm at a right angle with the arm, so that the palm of the hand will present to the patient's abdomen. Then seizing the wrist with one hand, and the surgical neck of the humerus with the other, while the arm is thus elevated and the forearm flexed, use the forearm as a lever, and rotate the head of the humerus upward, outward, and backward, until the palm of the patient's hand looks upward, and a strong resistance to further rotation, caused by the tendon of the subscapularis, is felt. Then bringing the elbow slowly to the side, and keeping the humerus parallel with the middle line of the axilla, that is, not carrying the arm either toward the anterior or posterior portion of the trunk, *rotate* the head of the humerus upward and forward by reversing the motion of the forearm until the palm of the hand shall again look downward, bringing the elbow to the side during this latter rotation, when the luxation will be reduced with great ease to both patient and surgeon.

To reduce an anterior luxation, carry the elbow as far backward as possible, and elevate it so as to throw the head of the bone into the axilla, then treat as in an original axillary dislocation. For a posterior luxation elevate the arm and carry it strongly forward, so as to make an axillary variety, and proceed as before.

The paper is illustrated by twelve cases, treated by this method, three being of the anterior variety, and the remainder axillary. One of the former was of three weeks' standing, and one of the latter of six weeks' duration. In about half the number, anæsthetic agents were not employed.

IX.—*On an Operation for Extracting a Stone from the Bladder by Urethrotomy and Dilatation of the Prostatic Urethra by means of a Dilating-Staff.* By MR. JOHN WOOD. (British Medical Journal, New Series, No. iii., 1861.)

The operation of Mr. Wood, practiced by him successfully in a boy, nine years of age, differs from the median method of Mr. Allarton, simply in the means employed to dilate the prostate and neck of the bladder, and in the size and form of the external incision. It is based upon the use of a staff opening at the curve into two blades, which admit between them, through an incision into the membraneous urethra, the forefinger of the operator, to effect forcible dilatation. The staff is furnished with a lever, pressure on which increases the dilating power, at the same time that the posterior blade is turned on its axis, and presses backward on the base of the bladder, thus holding it toward the perineum and preventing its yielding before the pressure of the finger. The preliminary incisions are of a lunated form, and are made with a very narrow-bladed knife, by commencing two lines to the right of the raphé, just behind the bulb, and carried in a curve to a point midway between the anus and left tuberosity of the ischium, terminating opposite to the former. By a divergence of the blades of the staff a groove is exposed, upon which the membraneous urethra is then to be opened from the bulb to the prostate gland, a little to the left of the median line, to avoid injuring the ejaculatory ducts. The prostate and neck of the bladder are then dilated by the finger, carried between the blades of the instrument.

The advantages of the dilating staff were: that the lateral tension renders a clean cut into the membraneous urethra, and a fair introduction of the finger to dilate, easier to accomplish; that the dilating finger is guided into the prostatic channel with the increased certainty of a conducting blade on each side, and is aided by the dilating action of the blades holding down the bladder, and preventing its yielding before the pressure of the finger, allowing it at the same time more tactile perception of the resisting tissues than can be obtained by the use of gorgets or dilators, which are also thus rendered unnecessary. The pressure of the separated blades guards against the passage of the finger between the bladder and pubes on the one hand, and the bladder and rectum on the other. Lastly, there is more positive certainty of avoiding section of the prostatic capsule, and exposure to the deleterious action of urine effused into the pelvic fascia above the levator ani, than can be obtained by the use of the knife or gorget in the prostate cutting in opposition to the contracting levator ani.

X.—*On Excision of the Head of the Femur.* By RICHARD BARWELL, F.R.C.S. (A Treatise on the Diseases of the Joints, p. 437. London: Churchill, 1861.)

The operation of removal of the head of the thigh-bone has attracted much attention and has been gaining in credit during the past fifteen years. In his admirable "Treatise on the Diseases of the Joints," Mr. Barwell gives, in the following terms, the best statistical accounts of its effects that we have yet seen:—

"I can thus gather 104 cases. Twelve times the operation was performed for injury, (eleven times gunshot injury, once for fracture of the thigh and descending ramus pubis.) Of these twelve but one recovered. Of the ninety-two cases in which joint disease was the cause of the operation, we find that fifty-six recovered, thirty-two are dead, four remain uncertain. Therefore, in eighty-eight cases, fifty-six recovered. Thus, the recoveries stand at the high ratio of 63·63 per cent. It must be, nevertheless, acknowledged that several of the patients, after having lived and even walked about for some months, or even more, ultimately succumbed to internal disease, generally to tuberculosis."

"Concerning the power or use of the limb afterward, it is necessary to speak with the greatest caution. Very many of the cases, after having been reported as cured, with perfect use of the limb, have been lost sight of just when the critical time for testing the use of the member has arrived. Many of these are, I believe, dead; others have not so much use in the limb as the first result of the operation might lead us to suspect. We may tabulate the only attainable numbers thus, but the quantity of 'useful limbs' is very much too high: of the fifty-six recoveries I get no reliable information in fourteen; in six the limb is useless, in thirty-six the limb is reported as useful."

